# Kyrieleis plaques in cytomegalovirus retinitis

**DOI:** 10.1007/s12348-011-0033-y

**Published:** 2011-08-12

**Authors:** Amar Patel, Matthew Pomykala, Krishna Mukkamala, Ronald C. Gentile

**Affiliations:** 1 Department of Ophthalmology, The New York Eye & Ear Infirmary, 310 East 14th Street, New York, NY 10003 USA; 2 Department of Ophthalmology, New York Medical College, Valhalla, NY USA

**Keywords:** Kyrieleis plaque, Cytomegalovirus, Retinitis, Segmental periarteritis

## Abstract

**Purpose:**

The purpose of this study is to report a case of Kyrieleis plaques (segmental retinal periarteritis) associated with cytomegalovirus (CMV) retinitis.

**Methods:**

A 47-year-old female with recently diagnosed human immunodeficiency virus and a CD4 count of 55 cells/µl presented with decreased vision and floaters in her left eye. Ophthalmic examination revealed an advancing border of white granular CMV retinitis extending into the macula. Intraocular aqueous specimen contained 420,000 copies/ml of CMV DNA by polymerase chain reaction. The patient was treated with intravitreal foscarnet and oral valganciclovir.

**Results:**

Kyrieleis plaques involving the retinal arteries were noted on presentation and increased during the first 6 weeks of treatment as the retinitis faded. The plaques on fluorescein angiography did not leak fluorescein dye and slowly faded over 5 months.

**Conclusions:**

Kyrieleis plaques can be seen in the setting of CMV retinitis. These plaques can be differentiated from vascular sheathing and frosted branch angiitis by its occurrence only in the retinal arteries and the absence of leakage of fluorescein dye.

## Introduction

Kyrieleis plaques, also referred to as segmental retinal periarteritis, were first described in an eye with tuberculosis uveitis by Kyrieleis in 1933 [1]. In 1959, Griffin and Bodian used the term segmental retinal periarteritis to describe them [2]. These plaques appear as whitish, segmented deposits found within the walls of the retinal arteries. Kyrieleis plaques have been primarily described in association with infections of the retina, *Toxoplasma gondii* chorioretinitis being the most common. They have also been reported with *Rickettsia conorii*, *Mycobacterium tuberculosis*, *Treponema pallidum*, and varicella-zoster virus (VZV) infections [1, 3–7].

The etiology of Kyrieleis plaques has not been well established. Orzalesi and Ricciardi suggested these lesions are an immune response to an infectious agent and result from the deposition of immune cells and inflammatory debris in the arterial walls [8]. Others have debated this hypothesis since these plaques can persist despite resolution of the infection and treatment with steroids [4]. Wise suggested these plaques represented arteriosclerosis, while others have postulated they resulted from migration of exudates from active choroiditis to periarterial sheaths where anatomical variation leads to compartmentalization of the exudates [2, 9]. No histopathological evaluation of these plaques has been performed.

The purpose of our report is to describe the presence of Kyrieleis plaques, distinct from vascular sheathing and frosted branch angiitis, associated with cytomegalovirus (CMV) retinitis. To our knowledge, this association appears to be rarely acknowledged and not previously reported.

## Case

**Fig. 1:**
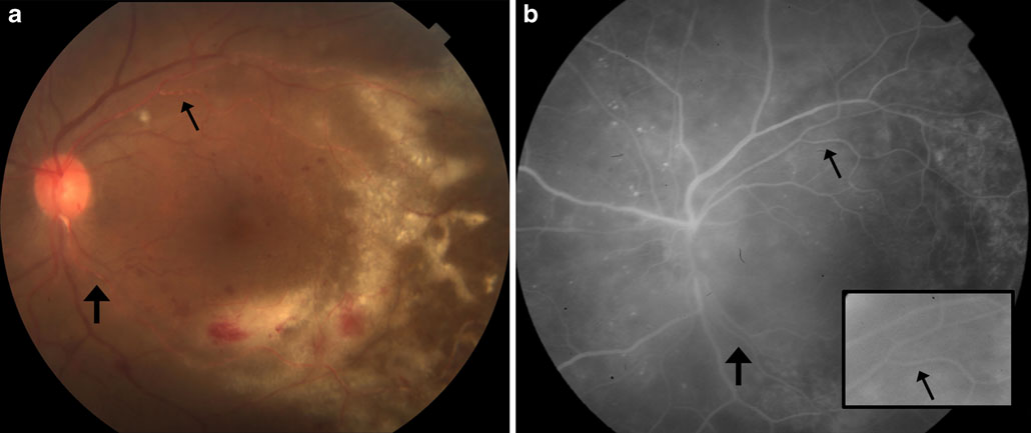
**a)** Fundus photo of the left eye with Kyrieleis plaques (*arrows*) involving the retinal arteries associated with an advancing border of granular Cytomegalovirus retinitis located at the border of the temporal and inferior macula. **b)** Fluoroescein angiography revealed normal arterial filling and absence of dye leakage from the Kyrieleis plaques (*arrows*). The *inset* represents a magnified view of the superior retinal artery at 5.5 minutes

A 47-year-old Hispanic female with untreated human immunodeficiency virus (HIV) presented with decreased vision and floaters in her left eye for 1-week duration. She was diagnosed with both HIV and type II diabetes mellitus 4 months prior. On ophthalmic examination, best-corrected visual acuity was 20/25 in the right eye and 20/60 in the left eye. The anterior chambers were deep and quiet, and the intraocular pressures were 18 mm Hg in both eyes. A few inflammatory cells were present in the vitreous of the left eye. Funduscopic examination revealed moderate non-proliferative diabetic retinopathy in both eyes and an advancing border of white granular retinitis surrounding the inferior and temporal macula of the left eye. Kyrieleis plaques were present on the retinal arteries without any significant sheathing or involvement of the retinal veins (Fig. 1a). Fluorescein angiography had normal retinal arterial filling with multiple hyperfluorescent microaneurysms and retinal pigment epithelial window defects peripheral to the leading edge of retinitis. Kyrieleis plaques seen on funduscopic examination did not leak or have significant late staining of fluorescein dye (Fig. 1b).

Laboratory evaluation revealed an elevated serum CMV IgG antibody with a negative systemic work up for *T. gondii*, VZV, herpes simplex virus (HSV), *T. pallidum*, and tuberculosis. CD4 T cell count was 55 cells/μL, and HIV viral load was 193,065 copies/ml. Polymerase chain reaction of the aqueous was positive for CMV DNA (420,000 copies/ml) and negative for HSV and *T. gondii* DNA.

**Fig. 2:**
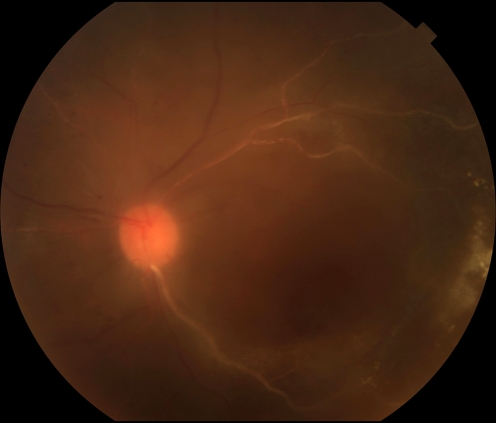
Fundus photo after 6 weeks of anti-viral treatment with a marked increase in Kyrieleis plaques as the CMV retinitis resolved

**Fig. 3:**
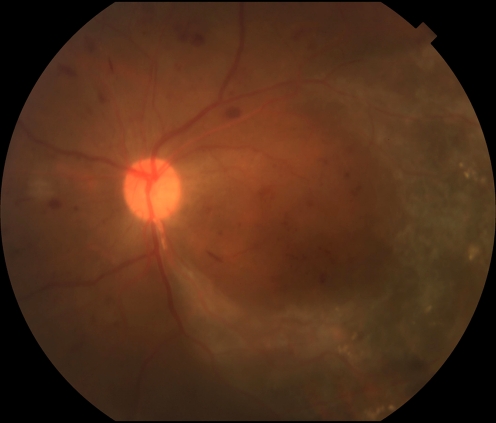
Fundus photo 5 months after initiation of treatment with fading of Kyrieleis plaques

The CMV retinitis was treated with intravitreal injections of foscarnet sodium and oral valganciclovir. The patient was also started on HAART, antiglycemic therapy, azithromycin for *Mycobacterium avium* complex prophylaxis, and atovaquone for *Pneumocystis jiroveci* pneumonia prophylaxis. Over the first 6 weeks as the retinitis resolved, the Kyrieleis plaques increased in number and become confluent along parts of the retinal arteries (Fig. 2). The Kyrieleis plaques subsequently faded over the next few months with a few persisting adjacent to the optic nerve at 5 months (Fig. 3). On follow-up, the CD4 T cell count increased to 305 cells/μL, and the HIV viral load became undetectable. Despite resolution of the retinitis, vision in the left eye decreased to 20/100, complicated by immune recovery uveitis and macular edema.

## Discussion

CMV retinitis typically presents with focal areas of retinal necrosis with primary lesions usually located adjacent to blood vessels, secondary to hematogenous spread of the virus [10]. On funduscopic examination, the appearance of CMV retinitis can range from a dry-appearing irregular and granular border with satellite lesions to an edematous and confluent area of thick, yellow-white necrosis associated with retinal hemorrhages and vascular sheathing [10].

Vascular involvement in CMV retinitis can result in vascular sheathing. Exudates around retinal vessels, more commonly veins, can result in focal areas of fluffy white cuffing or sheathing, with or without skip areas [11]. When the perivascular sheathing is severe, the retinal arteries and veins appear frosted, and the term “frosted branch angiitis” is used to describe this entity [12]. Kyrieleis plaques can be differentiated from vascular sheathing and frosted branch angiitis by its clinical and fluorescein angiographic features. Kyrieleis plaques affect only the retinal arteries in contrast to frosted branch angiitis that involves both the retinal arteries and veins. In addition, Kyrieleis plaques are confined to the vessel wall and do not leak fluorescein dye in contrast to frosted branch angiitis that extends outside the vessel wall and extensively leaks fluorescein dye [12, 13].

Kyrieleis plaques have been primarily reported in association with toxoplasmosis chorioretinitis. Although the cause of Kyrieleis plaques is unclear, the increase in these plaques following treatment and immune recovery in our case supports the theory of an immune response to an infectious agent and deposition of inflammatory debris as the etiology of these plaques. Kyrieleis plaques, although not specifically noted in many reports of infectious chorioretinitis, may be under reported. While many authors have concluded that Kyrieleis plaques are rare, Griffin and Bodian in 1959 felt that they may be more common than the literature suggests [2]. We have found at least one case of CMV retinitis in the literature that appears to also have had Kyrieleis plaques, similar to our patient [14]. Our case adds CMV retinitis to the list of causes of Kyrieleis plaques.
